# Comparison of the performance of HPV DNA chip test and HPV PCR test in cervical cancer screening in rural China

**DOI:** 10.3389/fmicb.2022.1040285

**Published:** 2022-11-11

**Authors:** Zhi-Fang Li, Xin-Hua Jia, Xin-Yu Ren, Bei-Ke Wu, Wen Chen, Xiang-Xian Feng, Li-Bing Wang, You-Lin Qiao

**Affiliations:** ^1^Department of Epidemiology, National Cancer Center/National Clinical Research Center for Cancer/Cancer Hospital, Chinese Academy of Medical Sciences and Peking Union Medical College, Beijing, China; ^2^Department of Preventive Medicine, Changzhi Medical College, Changzhi, China; ^3^State Key Laboratory of Molecular Vaccinology and Molecular Diagnostics, National Institute of Diagnostics and Vaccine Development in Infectious Diseases, Collaborative Innovation Center of Biologic Products, School of Public Health, Xiamen University, Xiamen, Fujian, China; ^4^School of Public Health, Shanxi Medical University, Taiyuan, China; ^5^Department of Pathology, Affiliated Heping Hospital of Changzhi Meidical College, Changzhi, China; ^6^Center for Global Health, School of Population Medicine and Public Health Chinese Academy of Medical Sciences and Peking Union Medical College, Beijing, China

**Keywords:** human papillomavirus, cervical cancer, HPV DNA chip test, HPV PCR test, screening

## Abstract

**Background:**

This study aimed to evaluate the performance of two different principles of HPV testing in primary cervical cancer screening and ASC-US triage in rural areas.

**Methods:**

3,328 and 3,913 women were enrolled in Shanxi, China in 2017 and 2018, respectively, and screened using liquid-based cytology and different HPV tests with a 4-year follow-up. Different screening methods commonly used in clinical practice were evaluated.

**Results:**

In the HPV PCR test cohort, the prevalence of HPV infection was 14.90%. A total of 38 cases of CIN2+ were identified at baseline, 2 of which were in the HPV-negative cohort and the rest in the HPV-positive cohort (2 = 186.85, *p* < 0.001). Fifty-three cases of CIN2+ were accumulated over 4 years. The HPV infection rate in the HPV DNA chip test cohort was 21.10%. A total of 26 CIN2+ cases were identified at baseline, all in the HPV-positive population (2 = 92.96, *p* < 0.001). 54 CIN2+ cases were cumulative over 4 years. At 4-year follow-up, HPV-negative results were significantly more protective against cervical intraepithelial neoplasia grade 2 or worse (CIN2+) than normal cytologic results at baseline. HPV screening was more sensitive and specific than cytologic screening (using ASC-US as the threshold) and performed better on the HPV DNA microarray test. In addition, compared with HPV 16/18 testing, sensitivity increases and specificity decreases when using HPV testing for cytologic ASC-US triage, regardless of which HPV test is used.

**Conclusion:**

In the rural areas where we implemented the study, HPV tests performed well for screening than LBC and HPV DNA chip testing performed better than HPV PCR testing in the screening cohort. Optimal screening was achieved technically when used in combination with LBC for ASC-US population triage, without thinking the feasibility for resource availability.

## Introduction

Cervical cancer is one of the top three most common malignancies among women worldwide. The Global Burden of Cancer 2020 report published by the International Agency for Research on Cancer of the World Health Organization showed that there were about 604,000 new cases of cervical cancer and 342,000 deaths worldwide in 2020; and there were about 110,000 new cases and 59,000 deaths in China, accounting for 18.2 and 17.3% of the global incidence and deaths, respectively. In addition, there was a trend toward younger age (Kang et al., 2014; Sung et al., 2021). Persistent infection with high-risk human papillomavirus (HR-HPV) is considered the leading cause of cervical cancer and precancerous lesions. HR-HPV infection can be detected in 60–70% and 20–40% of cervical lesions CIN2 to CIN3 and CIN1, respectively (Xu et al., 2016). More than 99% of patients with cervical cancer are detected with HPV infection in the cervical specimens (Sias et al., 2019). The 2020 guidelines recommended HPV testing as the primary screening tool for cervical cancers. However, in most developing countries (World Health Organization, 2021), such as China, the more widely used screening tool for cervical cancer is liquid-based cytology testing. HPV testing is gradually replacing cytology because it is fast and has high sensitivity and specificity. However, the feasibility of HPV screening test in resource-constrained areas need to be properly assessed (Banerjee et al., 2022).

The main kit technologies that have been marketed for HPV nucleic acid detection are the fluorescent PCR method, Hybrid Capture 2 (HC2), biochip, and flow-through fluorescent hybridization (Stoler et al., 2020). There is some variability in the results using different HPV DNA detection methods, which is mainly related to the principles of the detection methods and other aspects (Michelli et al., 2011). HPV DNA chip technology, a new HPV detection technology, has been evaluated in fewer studies. In this study, the real-time fluorescent PCR method was compared with the HPV DNA chip method. The aim was to compare the performance of two different HPV test kits using different screening strategies, that is, primary screening for cervical cancer and triage effect in ASC-US population.

## Materials and methods

### Study population

Two counties of Changzhi City, Shanxi Province (Changzhi county and Wuxiang County) were selected to establish cervical cancer screening cohorts. From May to June 2017, 3,328 rural women in Changzhi county underwent cervical cancer screening; From August to September 2018, 3,913 rural women in Wuxiang County underwent cervical cancer screening.

The inclusion criteria were as follows: (1) age ≥ 21 years with an intact uterus; (2) no history of cervical cancer treatment or cervical surgery; (3) no sexual intercourse within 48 h after screening and no vaginal medications, vaginal contraceptives, or vaginal washings within 48 h; (4) no suspected clinical pregnancy symptoms; pregnant women could participate in the study up to 8 weeks after the end of pregnancy; (5) signed informed consent.

### Study design and procedure

All study participants were included in the study after giving informed consent, completing a questionnaire and a gynecological examination. The questionnaires were administered by trained investigators and included general information about the study participants, such as marital status, education level, history of smoking and alcohol consumption, menstrual history and reproductive history.

A trained gynecologist performed gynecological examinations of the vulva, vagina, and cervix for each enrolled woman. Participants in Changzhi County had one cervical exfoliated cell collected by a gynecologist and preserved in cytology preservation solution (PreservCyt solution) for liquid-based cytology testing (LBC) and HPV DNA testing. Participants in Wuxiang County had two cervical samples collected by gynecologists: one cervical exfoliated cell was collected using a single-use cervical sampling swab and preserved in cytology preservation solution (PreservCyt solution) for LBC testing; one cervical sample (cervical exfoliated epithelial cells or secretions, etc.) was collected and preserved in cell preservation solution (Jiangsu Jiangyou Medical Technology Co. Ltd.) in a single-use sampling kit for HPV DNA testing.

**Figure 1:**
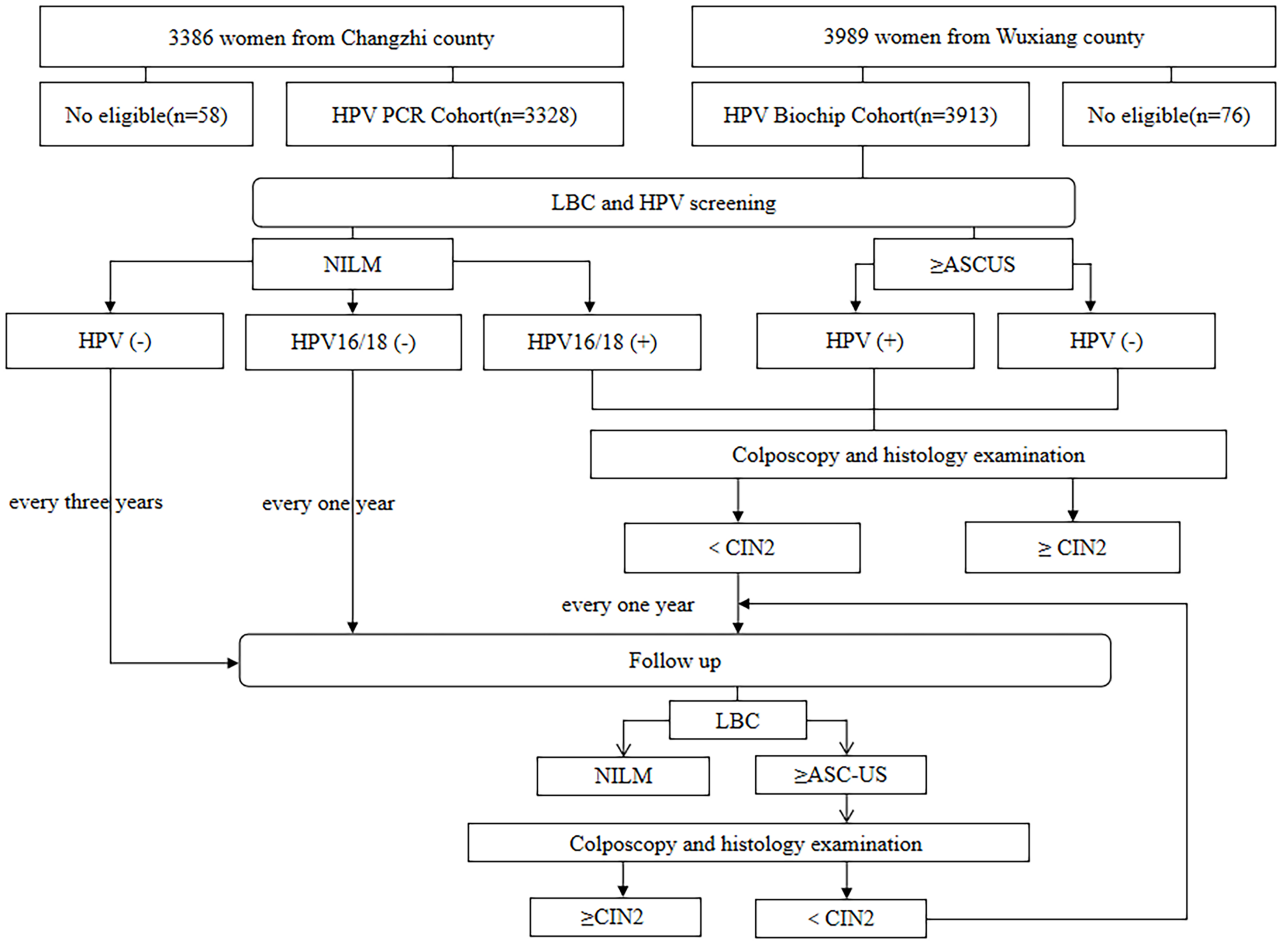
Flow chart of HPV PCR cohort and HPV biochip cohort.

Within 12 weeks of collection of cervical exfoliated cell specimens, those with positive results for HPV types 16 and/or 18 and cytology results ≥ASC-US were referred to undertake colposcopy by an experienced gynecologist. If the colposcopy result was abnormal, a cervical biopsy or endocervical curettage (ECC) was performed on the subject. Participants with HPV-negative test results and normal cytology results at baseline were followed up for cervical cytology after 3 years. In contrast, participants with HPV-positive test results or cytology ≥ASC-US at baseline received annual cervical cytology follow-up (3 years). Participants with ≥ASC-US results at follow-up received colposcopy, and participants with abnormal colposcopy received histopathology. During the study, participants with histopathological findings ≥CIN2 discontinued follow-up. as detailed in Figure 1.

Before program implementation, standard training was provided for cervical exfoliation cytology sampling (for cytology testing and HPV testing), stain production, reading, laboratory testing, colposcopy, and histopathology. All program staff were required to pass an assessment by the designated medical institution. Senior physicians from the Heping Hospital of Changzhi Medical College were on site for long periods of time to provide quality control guidance during the program.

### Laboratory tests

#### HPV DNA chip test

HPV typing nucleic acid detection kit (biochip method) (Beijing Bohui Innovation Biotechnology Group Co., Ltd.) uses a nucleic acid amplification-reverse spot hybridization technique combining PCR *in vitro* amplification and DNA reverse spot hybridization method to detect 14 (HPV 16, 18, 31, 33, 35, 39, 45, 51, 52, 56, 58, 59, 66, 68) high-risk human papillomavirus nucleic acid subtypes.

#### HPV PCR test

The HPV DNA typing test was performed using the high-risk HPV5 + 9 nucleic acid test kit (Tellgen Corporation). The test kit is based on fluorescent PCR technology and detects HPV 16, 18, 33, 52, 58, and 9 other HPV (31, 35, 39, 45, 51, 56, 59, 66, 68) typing for a total of 14 HPV and human genomic β-Globin DNA in a single reaction tube using a four-channel fluorescent PCR instrument with a minimum detection limit of 100 copies/ test, simultaneous detection of cervical exfoliated cell gene β-Globin.

#### Pathological diagnosis

All of the following tests and diagnostic procedures were strictly double-blinded. Cytology slides were read by two pathologists results and reported according to the Bethesda 2014 classification. The cytological results were as follows: negative for intraepithelial lesion or malignancy (NILM), atypical cells of undetermined significance (ASC-US), low-grade squamous intraepithelial lesion (LSIL), atypical squamous cells–cannot exclude high-grade squamous intraepithelial lesion (ASC-H), high-grade squamous intraepithelial lesion (HSIL), atypical glandular cells (AGC), and cervical cancer cells. Diagnoses were reported if the diagnoses by two cytologists were consistent. Otherwise, a third cytologist was consulted.

All participants with positive HPV 18 and/or HPV 16 testing or abnormal cytology (ASC-US or worse) were referred to undertake colposcopy. Two pathologists independently made diagnoses according to the 2014 WHO Classification of Tumors of the Female Genital Tract. If the diagnoses were concordant, they were reported as the pathologic diagnosis. Otherwise, a panel of pathologists was consulted to reach a consensual diagnosis. The histological diagnoses of cervical lesions were classified as normal, LSIL, HSIL/CIN2, HSIL/CIN3. HPV testing, cytology, and pathologic examination were performed with blinding to the results of each test. Participants with both negative HPV and cytology results were not referred for colposcopy and were considered normal.

### Statistical analysis

In this study, an ACCESS database was developed, double-entered and checked until complete consistency before analysis. Data were analyzed using R software (version 4.1.2). We reported numbers and percentages for categorical variables. Chi-square test were used to compare the difference between groups. *p* < 0.05(two-sided) was considered statistically significant. Using cervical intraepithelial neoplasia grade 2 or worse as a reference standard, sensitivity, specificity, positive predictive value, negative predictive value, and area under the receiver operating characteristic (ROC) curve (AUC) were calculated.

Based on a review of the published literature (Cox et al., 2013; Chatzistamatiou et al., 2016), we chose to evaluate six common clinical screening strategies. Strategy 1 used cytology as primary screening, referring participants with LBC ≥ ASC-US for colposcopy and participants with normal cytology for routine screening. Strategy 2 used HPV testing as primary screening and referred participants who were positive for HPV-16/18 for colposcopy. Strategies 3 and 4 used combined screening, with strategy 3 referring participants with ASC-US and HPV-16/18 positivity or LBC ≥ LSIL for colposcopy and strategy 4 referring participants with ASC-US and HPV positivity or LBC ≥ LSIL for colposcopy, and participants who did not undergo colposcopy were followed up at one-year or three-year intervals. Strategies 5 and 6 were to concurrently link cytology with HPV testing, i.e., strategy 5 referred participants for colposcopy if HPV 16/18 were positive or LBC ≥ ASC-US. Strategy 6 referred participants with HPV positivity or LBC ≥ ASC-US for colposcopy.

## Results

A total of 3,328 participants were included in the HPV PCR testing cohort, with an HPV prevalence of 14.90%. The majority of the participants were aged 40 years or above. About half of them completed secondary education, and almost all of them were married, non-smokers, and non-drinkers. There were no statistically significant differences in different subgroups of age at menarche, age at first pregnancy, number of pregnancies, and number of births.

A total of 3,919 participants were included in the HPV DNA chip testing cohort, with an HPV prevalence of 21.10%, and the distribution of population characteristics was similar to that of the HPV testing cohort and was overall comparable, as shown in Table 1.

**Table 1:** Population characteristics of different HPV testing cohorts at baseline.

Characteristic	HPV PCR test cohort (*N*, %)		*p*	HPV DNA chip test cohort (*N*, %)		*p*
	+	−		+	−	
Age			0.28			<0.01
21 ~ 29 years	12 (2.42)	109 (3.85)		26 (3.14)	104 (3.37)	
30 ~ 39 years	99 (19.96)	576 (20.34)		123 (14.87)	674 (21.84)	
≥40 years	385 (77.62)	2,147 (75.81)		678 (81.98)	2,308 (74.79)	
Education level			0.14			0.01
Primary School and below	116 (23.39)	577 (20.37)		270 (32.65)	850 (27.54)	
Middle School	227 (45.77)	1,344 (47.46)		357 (43.17)	1,344 (43.55)	
High School	89 (17.94)	455 (16.07)		78 (9.43)	269 (8.72)	
University and above	64 (13.90)	456 (16.10)		122 (14.75)	623 (20.19)	
Marital Status			0.11			0.01
Married	482 (97.18)	2,782 (98.23)		806 (97.46)	3,059 (99.13)	
Unmarried	14 (2.82)	50 (1.77)		21 (2.54)	27 (0.87)	
Smoking status			0.15			0.46
Never smoked	495 (99.80)	2,832 (100.00)		826 (99.88)	3,078 (99.74)	
Smoking	1 (0.20)	0 (0)		1 (0.12)	8 (0.26)	
Drinking status			0.03			0.01
Never drink	412 (83.06)	2,459 (86.83)		807 (97.58)	3,028 (98.12)	
Drink	84 (16.93)	373 (13.17)		20 (2.42)	58 (1.88)	
Age at menarche			0.32			0.01
≤14 years	271 (54.64)	1,616 (57.06)		374 (45.22)	1,602 (51.91)	
>14 years	225 (45.36)	1,216 (42.94)		453 (54.78)	1,484 (48.09)	
Age of first pregnancy			0.11			0.01
≤23 years	333 (67.14)	1795 (63.38)		479 (57.92)	1,510 (48.93)	
>23 years	163 (32.86)	1,037 (36.62)		348 (42.08)	1,576 (51.07)	
Number of pregnancies			0.85			0.13
≤3	394 (79.44)	2,260 (79.80)		527 (63.72)	2054 (66.56)	
>3	102 (20.56)	572 (20.20)		300 (36.28)	1,032 (33.44)	
Number of births			0.27			0.07
≤2	447 (90.12)	2,595 (91.63)		551 (66.63)	2,157 (69.90)	
>2	49 (9.88)	237 (8.37)		276 (33.37)	929 (30.10)	

Table 2 demonstrates the distribution of baseline and 4-year cumulative histopathological findings with different cytologic diagnoses at baseline and HPV status. In the population tested with HPV PCR, a total of 38 cases of CIN2+ were identified at baseline, 2 of which were found in the HPV-negative population and the rest in the HPV-positive population (*χ*^2^ = 186.85, *p* < 0.001). In the HPV DNA chip population, a total of 26 cases of CIN2+ were identified at baseline, all of which were found in the HPV-positive population (*χ*^2^ = 92.96, *p* < 0.001). Among the 4-year cumulative cases, a total of 53 cases of CIN2+ were found in the population with HPV PCR testing, 51 of which were in the HPV-positive population (*χ*^2^ = 274.36, *p* < 0.001). Fifty-four cases were found cumulatively in the HPV DNA chip population, all of which were in the HPV-positive population (*χ*^2^ = 199.55, *p* < 0.001), and all of these differences were statistically significant.

**Table 2:** Histopathological distribution in different cytological diagnoses regarding HPV status.

	HPV-negative results (*N*, %)				HPV-positive results (*N*, %)			
	No colposcopy	No CIN	CIN 1	CIN 2+	No colposcopy	No CIN	CIN 1	CIN 2+
*Baseline*								
HPV PCR test cohort								
NILM	2,478 (99.84)	2 (0.08)	2 (0.08)	0 (0.00)	305 (77.02)	51 (12.88)	23 (5.81)	17 (4.29)
ASC-US	82 (68.91)	28 (23.53)	8 (6.72)	1 (0.84)	16 (55.17)	8 (27.59)	2 (6.90)	3 (10.34)
ASC-H	0 (0.00)	25 (47.17)	27 (50.94)	1 (1.89)	5 (18.52)	7 (25.93)	8 (29.63)	7 (25.93)
AGC	2 (16.67)	9 (75.00)	1 (8.33)	0 (0.00)	1 (20.00)	1 (20.00)	3 (60.00)	0 (0.00)
LSIL	47 (34.31)	55 (40.15)	35 (25.55)	0 (0.00)	5 (20.83)	6 (25.00)	10 (41.67)	3 (12.50)
HSIL+	3 (10.34)	17 (58.62)	9 (31.03)	0 (0.00)	3 (20.00)	2 (13.33)	4 (26.67)	6 (40.00)
Total	2,612 (92.23)	136 (4.80)	82 (2.90)	2 (0.07)	335 (67.54)	75 (15.12)	50 (10.08)	36 (7.26)
HPV DNA chip test cohort								
NILM	2,874 (99.90)	3 (0.10)	0 (0.00)	0 (0.00)	597 (94.31)	24 (3.79)	8 (1.26)	4 (0.63)
ASC-US	66 (55.46)	53 (44.54)	0 (0.00)	0 (0.00)	46 (44.66)	32 (31.07)	19 (18.45)	6 (5.83)
ASC-H	2 (28.57)	4 (57.14)	1 (14.29)	0 (0.00)	4 (23.53)	9 (52.94)	2 (11.76)	2 (11.76)
AGC	0 (0.00)	1 (100.00)	0 (0.00)	0 (0.00)	0 (0.00)	0 (0.00)	0 (0.00)	1 (100.00)
LSIL	48 (59.26)	30 (37.04)	3 (3.70)	0 (0.00)	13 (24.07)	29 (53.70)	8 (14.81)	4 (7.41)
HSIL+	0 (0.00)	1 (100)	0 (0.00)	0 (0.00)	1 (5.26)	2 (10.53)	7 (36.84)	9 (47.37)
Total	2,990 (96.89)	92 (2.98)	4 (0.13)	0 (0.00)	661 (79.93)	96 (11.61)	44 (5.32)	26 (3.14)
*4-year cumulative*								
HPV PCR test cohort								
NILM	2,476 (99.84)	2 (0.08)	2 (0.08)	0 (0.00)	300 (77.32)	48 (12.37)	22 (5.67)	18 (4.64)
ASC-US	82 (68.91)	28 (23.53)	8 (6.72)	1 (0.84)	16 (45.71)	8 (22.86)	2 (5.71)	9 (25.71)
ASC-H	0 (0.00)	25 (47.17)	27 (50.94)	1 (1.89)	4 (14.81)	6 (22.22)	8 (29.63)	9 (33.33)
AGC	2 (16.67)	9 (75.00)	1 (8.33)	0 (0.00)	0 (0.00)	1 (25.00)	1 (25.00)	2 (50.00)
LSIL	47 (34.31)	55 (50.15)	35 (25.55)	0 (0.00)	5 (19.23)	6 (23.08)	9 (34.62)	6 (23.08)
HSIL+	3 (10.34)	17 (58.62)	9 (31.03)	0 (0.00)	3 (18.75)	2 (12.50)	4 (25.00)	7 (43.75)
Total	2,610 (92.23)	136 (4.81)	82 (2.90)	2 (0.07)	328 (66.13)	71 (14.31)	46 (9.27)	51 (10.28)
HPV DNA chip test cohort								
NILM	2,874 (99.90)	3 (0.10)	0 (0.00)	0 (0.00)	585 (94.35)	22 (3.55)	8 (1.29)	5 (0.81)
ASC-US	66 (55.46)	53 (44.54)	0 (0.00)	0 (0.00)	46 (43.81)	30 (28.57)	15 (14.29)	14 (13.33)
ASC-H	2 (28.57)	4 (57.14)	1 (14.29)	0 (0.00)	3 (12.50)	9 (37.50)	1 (4.17)	11 (45.83)
AGC	0 (0.00)	1 (100)	0 (0.00)	0 (0.00)	0 (0.00)	0 (0.00)	0 (0.00)	1 (100.00)
LSIL	48 (59.26)	30 (37.04)	3 (3.70)	0 (0.00)	13 (23.64)	26 (47.27)	7 (12.73)	9 (16.36)
HSIL+	0 (0.00)	1 (100.00)	0 (0.00)	0 (0.00)	1 (4.55)	2 (9.09)	5 (22.73)	14 (63.64)
Total	2,990 (96.89)	92 (2.98)	4 (0.13)	0 (0.00)	648 (78.36)	89 (10.76)	36 (4.35)	54 (6.53)

In the two cohorts based on different HPV detection methods, the specificity was better, around 90%, if LBC alone was used as a screening strategy, but the sensitivity was poorer and there was a more severe underdiagnosis. And the difference in positive predictive values for baseline CIN2+ and 4-year cumulative CIN2+ was not significant. If strategy 2 is implemented, that is, colposcopy in HPV16/18 positive population, the sensitivity reached around 80% and HPV detection based on HPV DNA chip technology is superior to conventional PCR technology with a sensitivity of 84.62 (95% *CI*: 64.27, 94.95) and 77.36 (95% *CI*: 63.45, 87.27), respectively. There was a relatively good improvement in sensitivity and specificity when LBC was combined with HPV testing for screening. The sensitivity of both strategies 3 and 4 decreased when using CIN2+ as the gold standard. However, precancerous lesions are a progressive state. Therefore, there was a significant improvement in sensitivity and specificity when using the 4-year cumulative CIN2+ as the gold standard. For strategies 3, 4, 5 and 6, HPV DNA chip technology performed better than PCR HPV test. The same trend was shown for CIN2+ over the next 4 years (Table 3).

**Table 3:** The sensitivity (SE), specificity (SP), positive predictive value (PPV) and negative predictive value (NPV) of various assays for CIN2+ endpoints.

Screening strategy		SE (%, 95CI)	SP (%, 95CI)	PPV (%, 95CI)	NPV (%, 95CI)
*HPV PCR test cohort*					
CIN2+ at baseline					
	Strategy 1	55.26 (38.47, 71.01)	86.96 (85.75, 88.08)	4.67 (2.98, 7.15)	99.40 (99.03, 99.64)
	Strategy 2	76.32 (59.39, 87.97)	96.47 (95.77, 97.07)	20.00 (14.01, 27.62)	99.72 (99.44, 99.86)
	Strategy 3	52.63 (36.05, 68.69)	91.19 (90.15, 92.12)	6.45(4.09, 9.94)	99.40 (99.04, 99.64)
	Strategy 4	52.63 (36.05, 68.69)	90.55 (89.48, 91.51)	6.04 (3.83, 9.33)	99.40 (99.03, 99.63)
	Strategy 5	97.37 (84.57, 99.86)	84.07 (82.77, 85.30)	6.60 (4.75, 9.06)	99.96 (99.77, 100.00)
	Strategy 6	100.00 (88.57, 100.0)	75.44 (73.93, 76.90)	4.49(3.24, 6.17)	100.00 (99.80, 100.00)
4-year cumulative CIN2+					
	Strategy 1	66.04 (51.64, 78.11)	87.08 (85.88, 88.20)	7.64 (5.45, 10.57)	99.37 (98.99, 99.62)
	Strategy 2	77.36 (63.45, 87.27)	96.67 (95.98, 97.25)	27.33 (20.54, 35.32)	99.62 (99.32, 99.80)
	Strategy 3	80.77 (60.02, 92.69)	94.75 (93.99, 95.42)	9.33 (6.01, 14.10)	99.86 (99.66, 99.95)
	Strategy 4	84.62 (64.27, 94.95)	93.26 (92.41, 94.01)	7.75 (5.03, 11.65)	99.89 (99.70, 99.96)
	Strategy 5	98.11 (88.62, 99.90)	84.27 (82.97, 85.50)	9.17 (6.98, 11.93)	99.96 (99.77, 100.00)
	Strategy 6	100.00 (91.58, 100.0)	75.79 (74.27, 77.24)	6.26 (4.77, 8.17)	100.00 (99.81, 100.00)
*HPV DNA chip test cohort*					
CIN2+ at baseline					
	Strategy 1	84.62 (64.27, 94.95)	90.20 (89.21, 91.11)	5.46 (3.53, 8.27)	99.89 (99.69, 99.96)
	Strategy 2	84.62 (64.27, 94.95)	94.88 (94.13, 95.54)	9.95 (6.48, 14.87)	99.89 (99.70, 99.97)
	Strategy 3	60.38 (46.02, 73.24)	91.33 (90.30, 92.26)	10.13 (7.13, 14.12)	99.30 (98.92, 99.56)
	Strategy 4	64.15 (49.75, 76.51)	90.69 (89.63, 91.65)	10.03 (7.14, 13.85)	99.36 (98.99, 99.61)
	Strategy 5	100.0 (83.98, 100.00)	87.03 (85.93, 88.07)	4.91 (3.29, 7.20)	100.00 (99.86, 100.00)
	Strategy 6	100.0 (83.98, 100.00)	74.02 (72.60, 75.38)	2.51 (1.68, 3.71)	100.00 (99.83, 100.00)
4-year cumulative CIN2+					
	Strategy 1	90.74 (78.94, 96.54)	90.49 (89.51, 91.39)	11.78 (8.92, 15.36)	99.86 (99.65, 99.95)
	Strategy 2	72.22 (58.14, 83.14)	95.26 (94.53, 95.90)	17.57 (12.93, 23.36)	99.59 (99.31, 99.76)
	Strategy 3	83.33 (70.21, 91.64)	95.08 (94.33, 95.73)	19.15 (14.44, 24.89)	99.76 (99.52, 99.88)
	Strategy 4	92.45 (80.93, 97.55)	93.58 (92.74, 94.32)	16.50 (12.56, 21.33)	99.89 (99.70, 99.96)
	Strategy 5	98.15 (88.82, 99.90)	87.43 (86.34, 88.45)	9.85(7.53, 12.76)	99.97 (99.81, 100.00)
	Strategy 6	100.00 (91.73, 100.0)	74.55 (73.14, 75.92)	5.21 (3.97, 6.79)	100.00 (99.83, 100.00)

**Figure 2:**
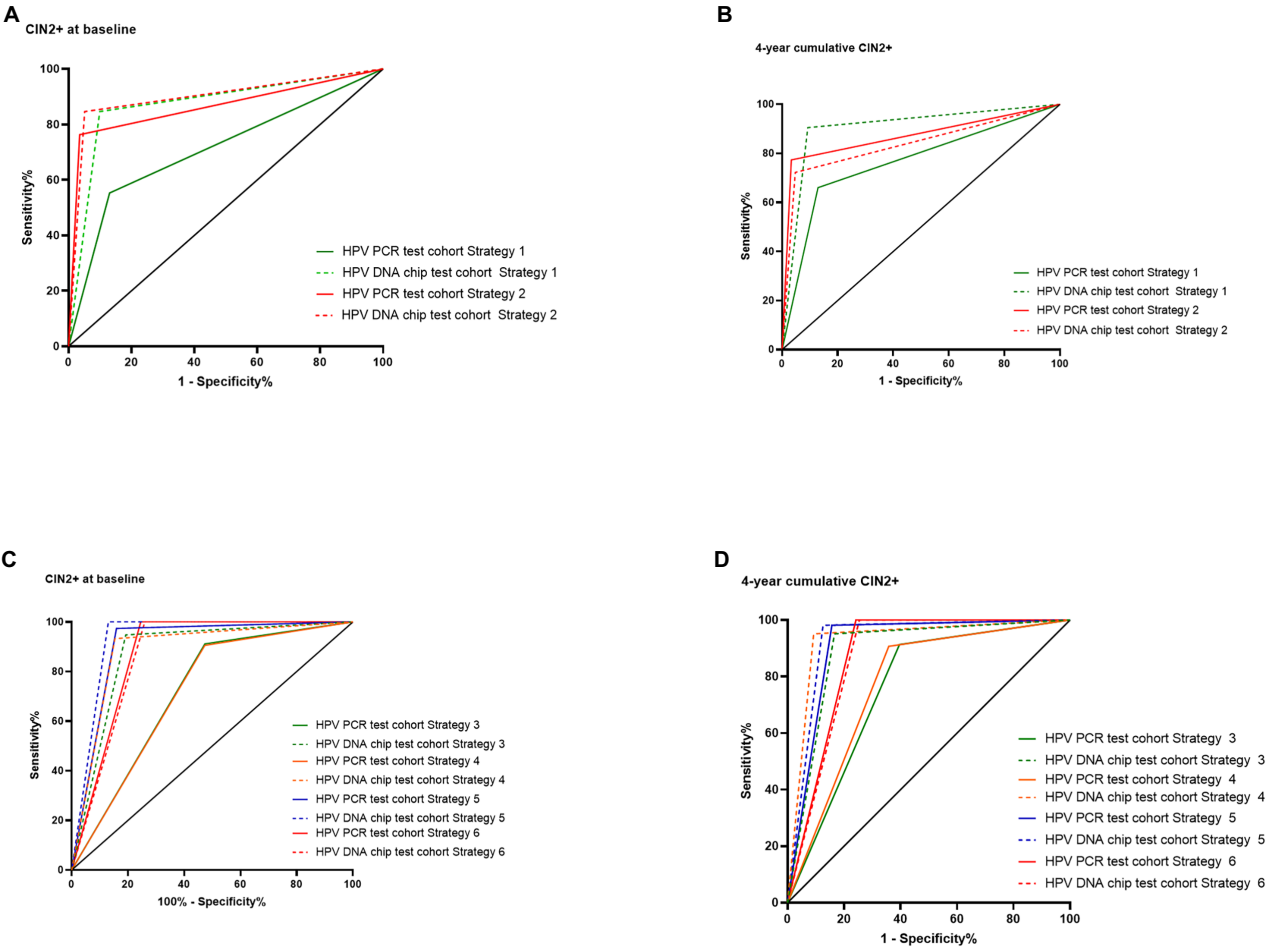
ROC curves of HPV DNA Chip and PCR testing techniques in different screening strategies.

Figure 2 shows the ROC curves plotted for different screening strategies. When using cytology only as a screening method (strategy 1), the AUC of HPV PCR assay = 0.71, 0.77, respectively, at baseline and 4 year cumulative, which are much lower than HPV Chip assay = 0.87, 0.91, respectively. as shown in A and B. C and D indicate ROC curves for strategies 3, 4, 5, and 6 at baseline and 4-year cumulative, respectively. HPV testing as a primary screening method or for ASC-US triage, and it can be concluded that when using CIN2+ as an endpoint, LBC with HPV chip test combined screening can achieve the maximum AUC (0.93) at baseline, for 4 years of cumulative CIN2+ as well.

## Discussion

The aim of cervical cancer screening is to detect and treat precancerous lesions early before they occur. In addition, women who participated in organized cervical cancer screening had a 41–92% reduction in cervical cancer mortality (Jansen et al., 2020) and a 50–60% reduction in all-cause mortality (Arbyn et al., 2012) compared to women who did not participate in screening. Therefore, cervical cancer screening remains an effective strategy for the prevention and control of cervical cancer (Simms et al., 2016). In rural areas, the lack of health resources is one of the most challenging factors. Measures to improve screening efficiency, reduce missed diagnoses, and decrease colposcopy referrals can improve health resource utilization. Combining cytology with HPV can improve screening in areas with poor health resources (Mezei et al., 2017; Jansen et al., 2021). Numerous studies have shown that HPV-based cervical screening is more effective than cytology alone (Balasubramanian et al., 2010; Ronco et al., 2014; Schiffman et al., 2015; Zhao et al., 2020). However, increased colposcopy referral rates are a potential problem with HPV-based screening (Zhao et al., 2021), whereas cytology has the inherent advantage of high specificity (Thomsen et al., 2021). In addition, it remains controversial whether combination testing or HPV testing alone should be used as the primary screening tool (Castle et al., 2011; Cox et al., 2013; Blatt et al., 2015; Cuschieri et al., 2018; Schiffman et al., 2018; Chan et al., 2020). Therefore, this study evaluated two testing methods by comparing primary screening for HPV 16/18 and triage for the ASC-US population.

In this study, we analyzed two large cohorts of women with baseline screening and 4-year histological follow-up. Both cohorts in this study were from resource-constraint areas and had similar demographic characteristics, so they were comparable. In the PCR cohort, 51 (1.53%) CIN2+ cases were found cumulatively over 4 years, while in the chip cohort, 54 (1.38%) cases were found cumulatively over 4 years, with no statistically significant differences. In the present study, DNA microarray outperformed PCR performance. The DNA microarray performed better when LBC was combined with HPV screening, both for baseline CIN2+ and cumulative CIN2+ over 4 years. Differences in detection principles may be an important reason for this difference. The sensitivity of LBC (ASC-US+) in the two cohorts in this study was 55.26 and 84.62%, respectively. The difference in cytology sensitivity between the PCR and chip cohorts may be related to the performance of cytology physicians and field sampling, and it is important to note that these physicians were from low to moderate resource areas.

In a previous study that pooled 40 studies, it showed that different principles of HPV testing perform differently in screening (Koliopoulos et al., 2017). HC2 is the conventional HPV DNA detection technique. For CIN 2+, the pooled sensitivity of HC2 and LBC (ASC-US+) was estimated to be 89.9 and 72.9%, and the pooled specificity was estimated to be 89.9 and 90.3%. The sensitivity of LBC (ASC-US+) was generally between 52 and 94% (Malloy et al., 2000; Tsiodras et al., 2010). The sensitivity of PCR ranged from 75 to 100% and the specificity ranged from 85 to 97%, which is overall consistent with the performance of this study. In most studies (An et al., 2003; Lee et al., 2005; Inoue et al., 2006; Jun et al., 2015), the HPV DNA chip test was used primarily for HPV genotyping.

The advantage of this study is the ability to see the long-term effects of screening through 4-year follow-up. Cytology sampling and diagnosis are from rural areas, which more realistically reflects the performance of cytology in areas with poor and moderate resources, but this is also a disadvantage. In addition, the cytology performance of the two cohorts was inconsistent, which may be related to the inconsistent performance of physicians in the regions or because of differences in population characteristics. Thirdly, as colposcopy was not performed in our programme in the cytologically normal and HPV-negative population, this may have led to partial under-diagnosis. Furthermore, colposcopic images were not reviewed and the subjectivity of the colposcopist can influence sampling. Finally, health resources and demographic characteristics vary widely across different provinces in China. Also, the cohort population was from two counties in Shanxi province, which is not fully representative of rural China. Therefore, the results of this study need to be further validated in other rural areas.

More studies should be conducted in the future to confirm the performance of HPV DNA chip. The development of HPV DNA chip with low price and high performance could provide more options for HPV test-based cervical cancer screening.

## Conclusion

In conclusion, we selected two rural areas in China for a four-year follow-up to evaluate cytology and different HPV testing principles and concluded that HPV testing is more effective than LBC for screening and HPV DNA microarray testing is more effective than HPV PCR testing. Optimal screening can be achieved when used in combination with LBC for ASC-US population triage, if consideration is given to the feasibility of resource availability.

## Data availability statement

The original contributions presented in the study are included in the article/supplementary material, further inquiries can be directed to the corresponding author.

## Ethics statement

The studies involving human participants were reviewed and approved by Affiliated Heping Hospital of Changzhi Meidical College. The patients/participants provided their written informed consent to participate in this study.

## Author contributions

Y-LQ and X-XF contributed to the design. Z-FL and X-HJ wrote the manuscript. B-KW, X-YR, and WC performed statistical analysis and HPV test. L-BW contributed to cytology and histology examination. All authors read and approved the final manuscript.

## Funding

This research was supported by CAMS Innovation Fund for Medical Sciences (no. CAMS 2021-I2M-1-004).

## Conflict of interest

The authors declare that the research was conducted in the absence of any commercial or financial relationships that could be construed as a potential conflict of interest.

## Publisher’s note

All claims expressed in this article are solely those of the authors and do not necessarily represent those of their affiliated organizations, or those of the publisher, the editors and the reviewers. Any product that may be evaluated in this article, or claim that may be made by its manufacturer, is not guaranteed or endorsed by the publisher.
